# Supplementary material for: Tumor‐stroma interactions differentially alter drug sensitivity based on the origin of stromal cells
Source: Mol Syst Biol. 2018 Aug 6;14(8):e8322. doi: 10.15252/msb.20188322 (PMC6078165; doi:10.15252/msb.20188322)
Supplement: Supplementary file 1 — Appendix [file MSB-14-e8322-s001.pdf]

APPENDIX

Tumor-Stroma Interactions Differentially Alter Drug Sensitivity

Based on the Origin of Stromal Cells

Benjamin D. Landry, Thomas Leete, Ryan Richards, Peter Cruz-Gordillo, Hannah R. Schwartz, Megan E. Honeywell, Gary Ren, Alyssa D. Schwartz, Shelly R. Peyton and Michael J. Lee

Table of contents

Appendix Figures S1-S11 p. 2-12

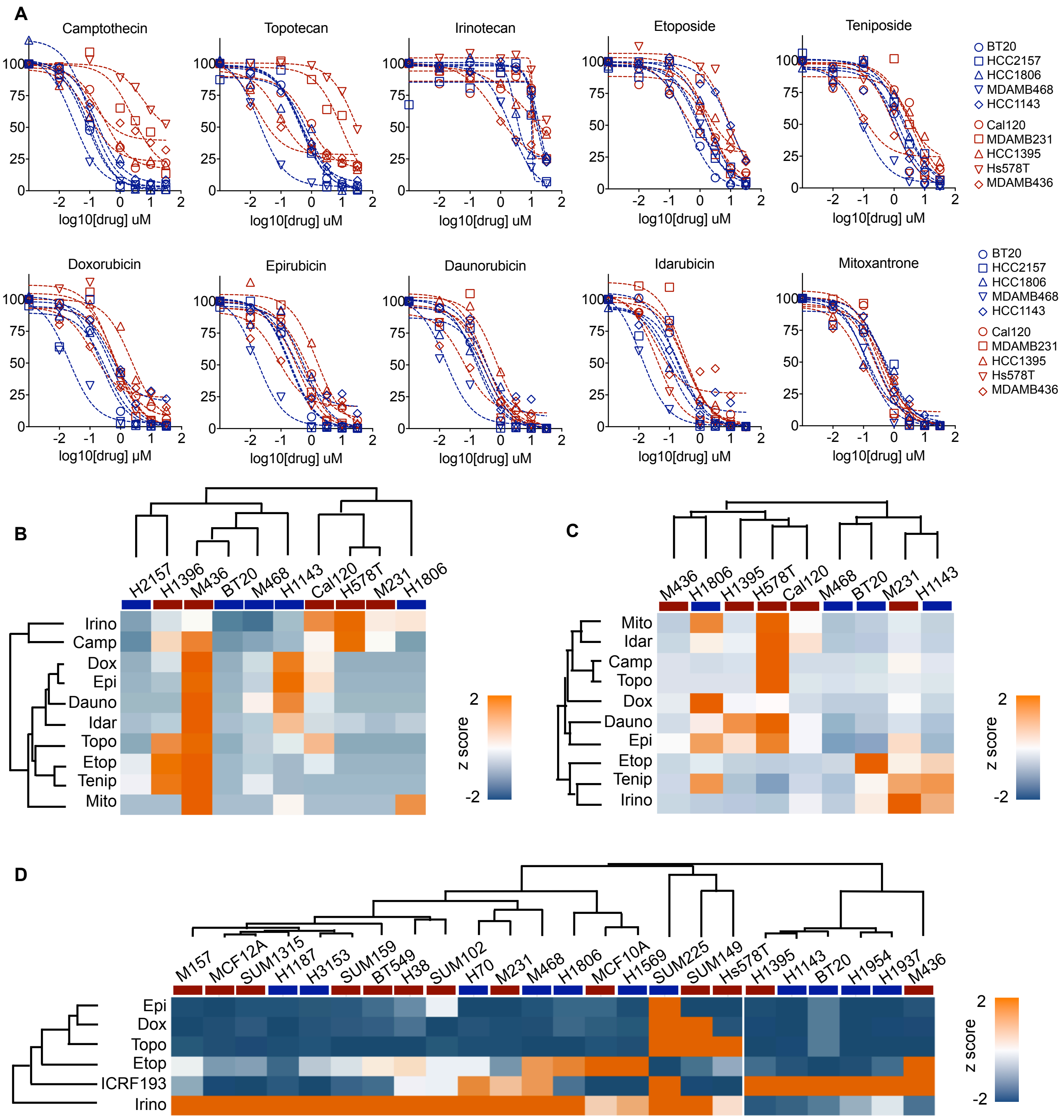

Appendix Figure S1: Variable sensitivity to common DNA damaging agents in TNBC cells. (A) Drug dose response curves for 10 Topo I and Topo II inhibitors. Data are presented as in Figure 1A. **(B)** Cell viability measured as in (A) for 10 Topo I/II inhibitors. Data are z-scored max death at 72 hours (Emax). Dendograms from hierarchical clustering shown for drugs and for cells **(C)** Hierarchical clustering, as in panel B, but for TNBC cell lines grown in 3D spheroids in matrigel. Data are EC50s as in Figure 1B. Nine TNBC cell lines shown. HCC-2157 did not grow in 3D. **(D)** EC50 for 24 TNBC cell lines (11 BL; 13 ML) treated with 6 Topo inhibitors. Data are from the LINCS consortium, as in Figure 1C. As with data in (B), hierarchical clustering fails to separate BL and ML cells. Additionally neither of the two major clades are enriched for BL or ML cells (p = 0.2393 for smaller clade on right; p = 0.9726 for larger clade on left). For panels B-D, basal-like cells are highlighted with a blue bar; mesenchymal-like cells are highlighted with a red bar.

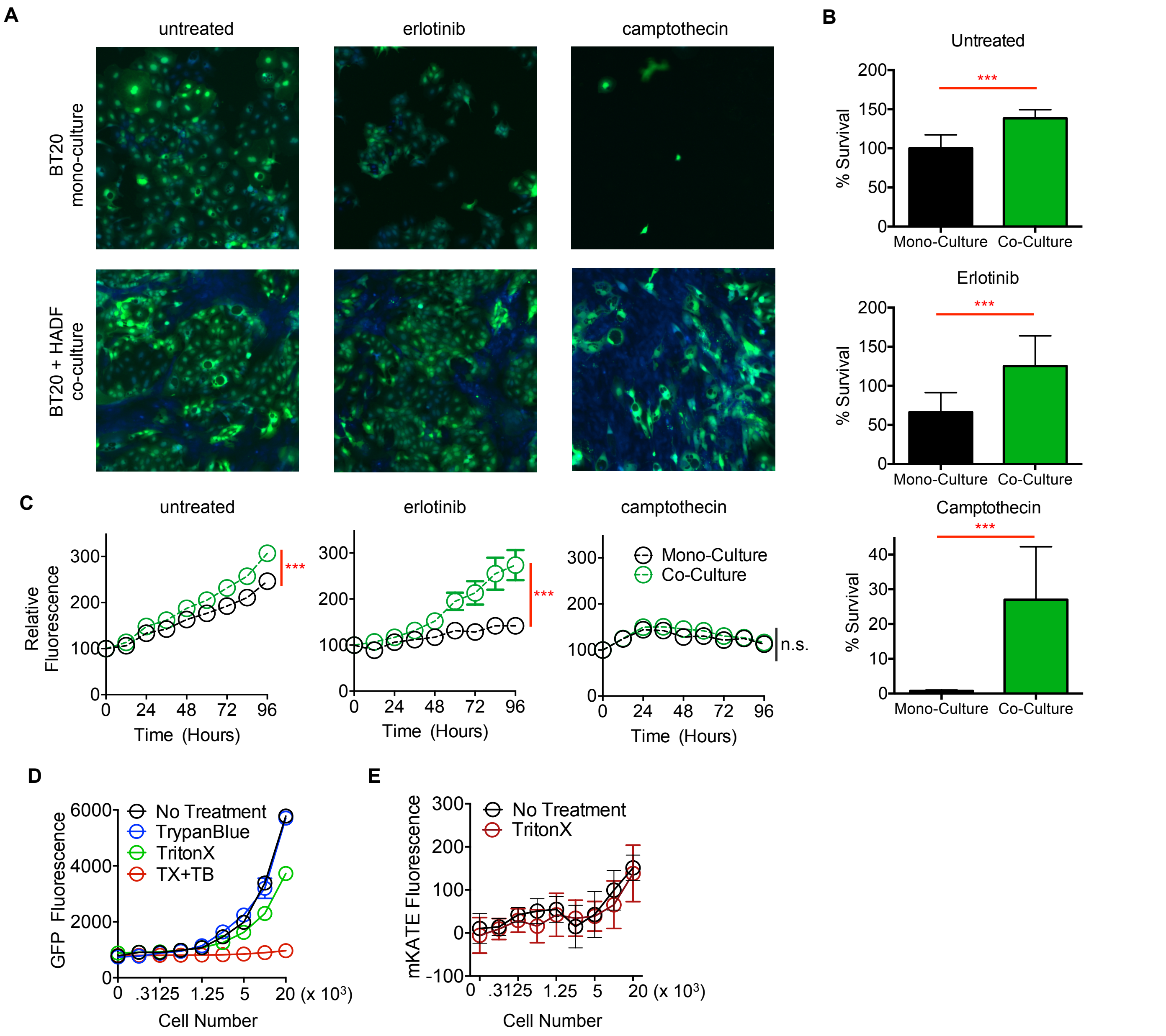

Appendix Figure S2: GFP-based measurements do not accurately report cell death. (A) Cell viability based on quantitative image analysis. Mono-culture is BT-20 cells expressing GFP; co-culture is GFP-BT20 + HADF, seeded at a 1:1 ratio. Images collected using fluorescence microscopy following a 96-hour exposure to 10 μM erlotinib or 500 nM camptothecin. GFP-BT20 cells are green; HADF are stained blue using a whole cell stain. **(B)** Quantitative analysis of images from experiment described in panel A. Analysis performed using CellProfiler. % Survival is relative to untreated cells grown in mono-culture. Eight images collected per condition from four biological replicates (2 images from each well). At least 300 cells were counted in every image, except BT-20 mono-cultures treated with camptothecin, where the average number of cells per image was 20. **(C)** Quantification of cell number using GFP fluorescence measured via fluorescence plate reader. Experimental samples are the same as in panels (A/B) but data were collected using a fluorescence plate reader at 12-hour intervals. Data are from biological quadruplicate experiments. Well-based fluorescence measurements taken using a plate reader accurately capture proliferation related phenotypes but fail to capture differences in the degree of cell death. Statistics for panels B and C were performed using t-test; *** = $p<0.05$; n.s. = not significant. **(D)** Titration of GFP sensitivity in a fluorescence plate reader. GFP fluorescence was quantified from a serial 2-fold dilution of GFP- BT20 cells using a plate reader. Fluorescence above background was seen for wells containing >1250 cells. Trypan Blue (which quenches GFP but does not diffuse into healthy cells) did not affect GFP fluorescence. 0.2% Triton-X was used to disrupt cell membrane integrity (no cells were visible by microscopy after 20 minutes), but GFP fluorescence was maintained. Triton-X + Trypan Blue reduced well fluorescence to background levels, suggesting that GFP remains stable and fluorescent after cells have died. Measurements taken 8 hours after exposure to TX or TB. **(E)** Titration as in panel (D) but using mKATE expressing cells. As with GFP, mKATE fluorescence is not changed 8 hours after cells are lysed with Triton-X.

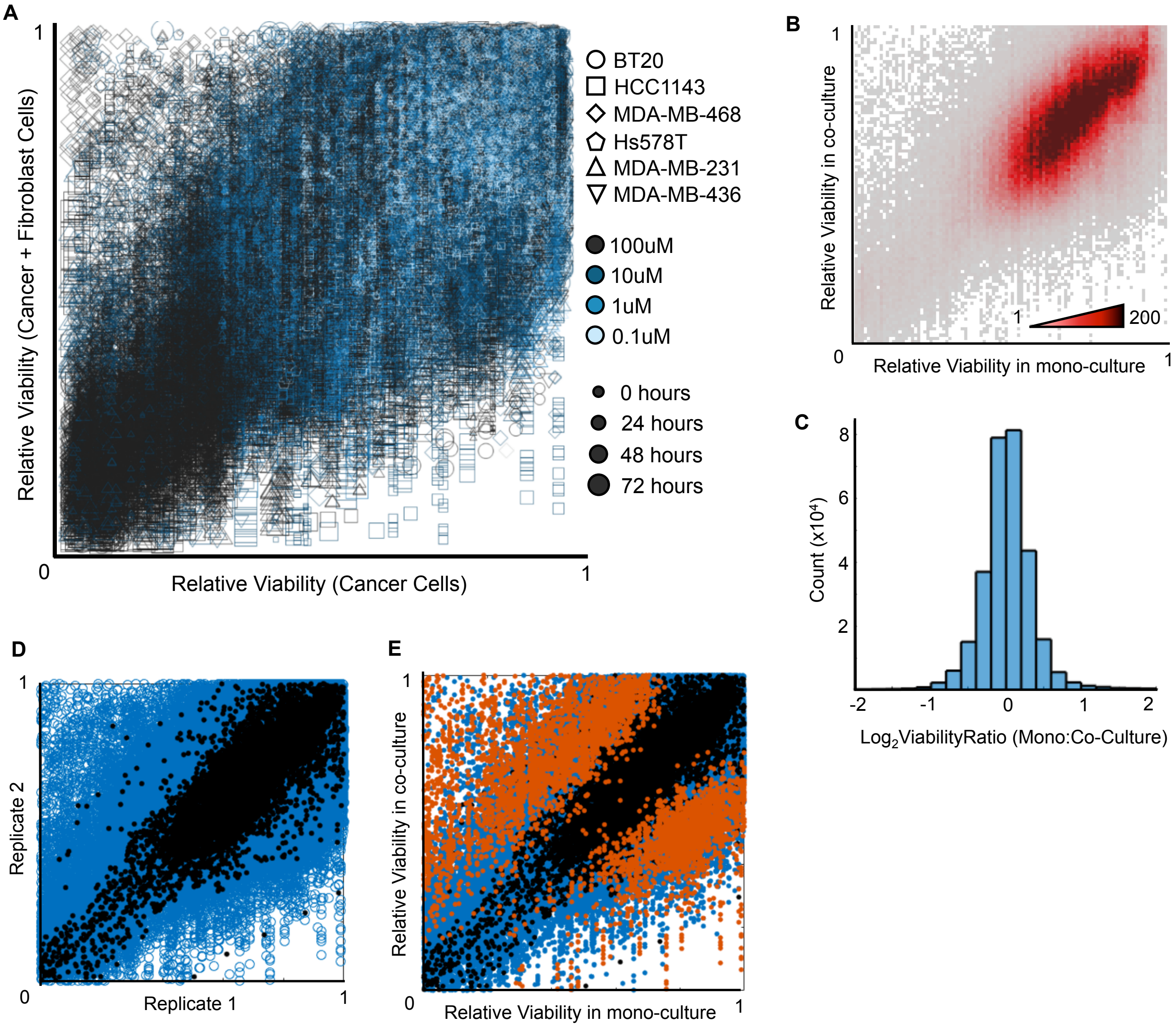

Appendix Figure S3: Statistical analysis of co-culture screen to identify TNBC-fibroblast interactions that significantly alter drug sensitivity. (A) Scatterplot of co-culture drug screen, as in Figure 2E, but with cell line, dose, and time highlighted. **(B)** Density plot of co-culture screen data. 95% of 312,120 data points fall within a single dense cluster (20-50% death; no influence of fibroblast). Scale bar shows density scale (i.e. the number of unique tumor-fibroblast-drug data points that fall within that region of the scatter plot). **(C)** Ratio of drug response in mono-culture vs. co-culture with primary fibroblasts. Data are normally distributed. **(D)** Correlation among replicates (r2 = 0.7315). Two biological replicates of mono-culture drug response are shown in black (total dataset in blue, shown as reference). **(E)** TNBC-fibroblast interactions that significantly alter drug response shown in orange (z score of mono:co-culture ratio > 3). 5039 drug responses were significantly altered relative to error among control replicates (black). See also Table 3 and Table 4.

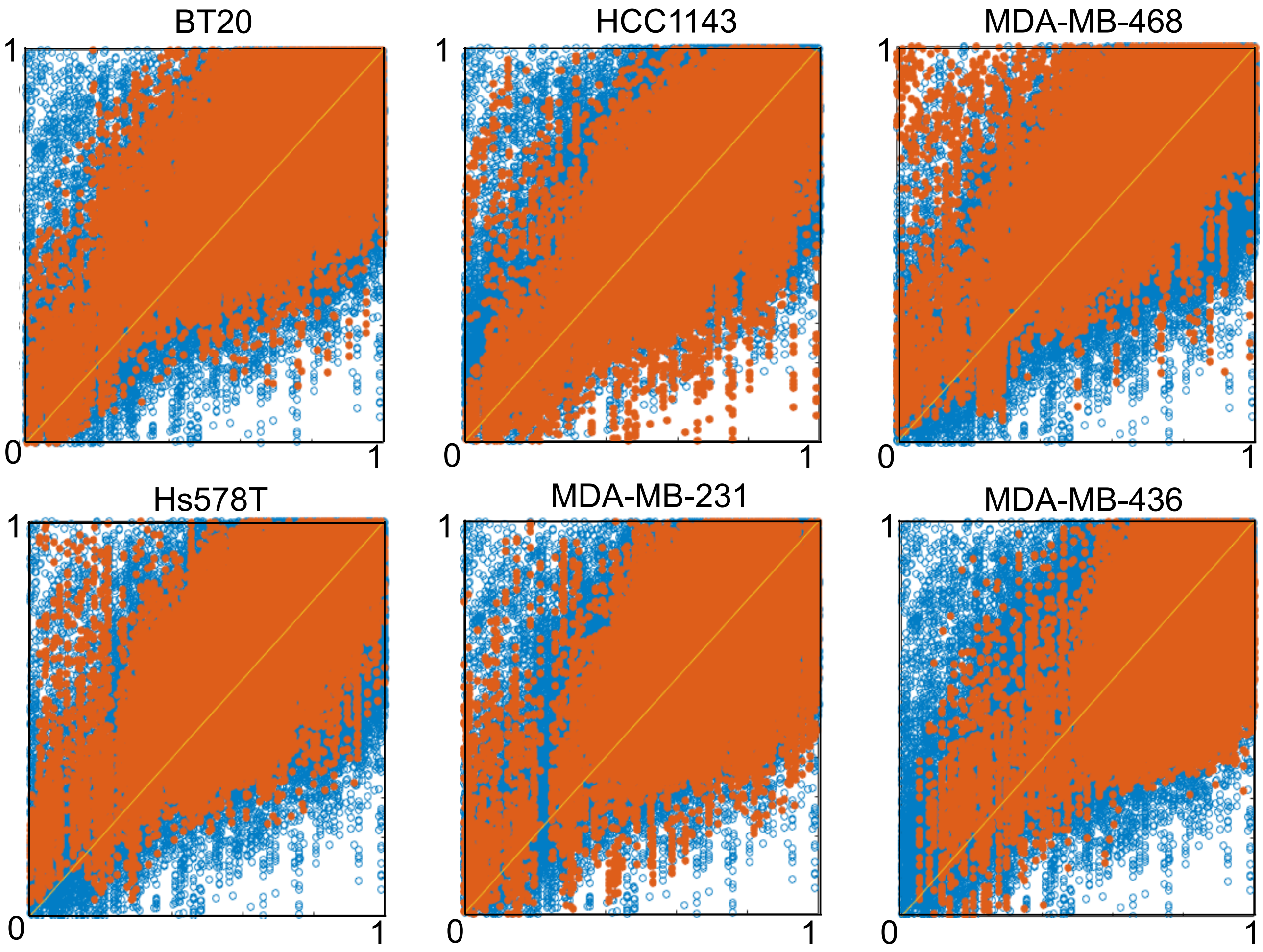

Appendix Figure S4: Fibroblasts influence drug sensitivity across TNBC cell lines. Data plotted as in Figure 2E, with drug responses of TNBC cells grown in mono-culture on the x-axis, and responses in co-culture with CAFs on the y-axis. In each plot, the overall dataset is shown in blue and the data for each TNBC cell line is shown in orange.

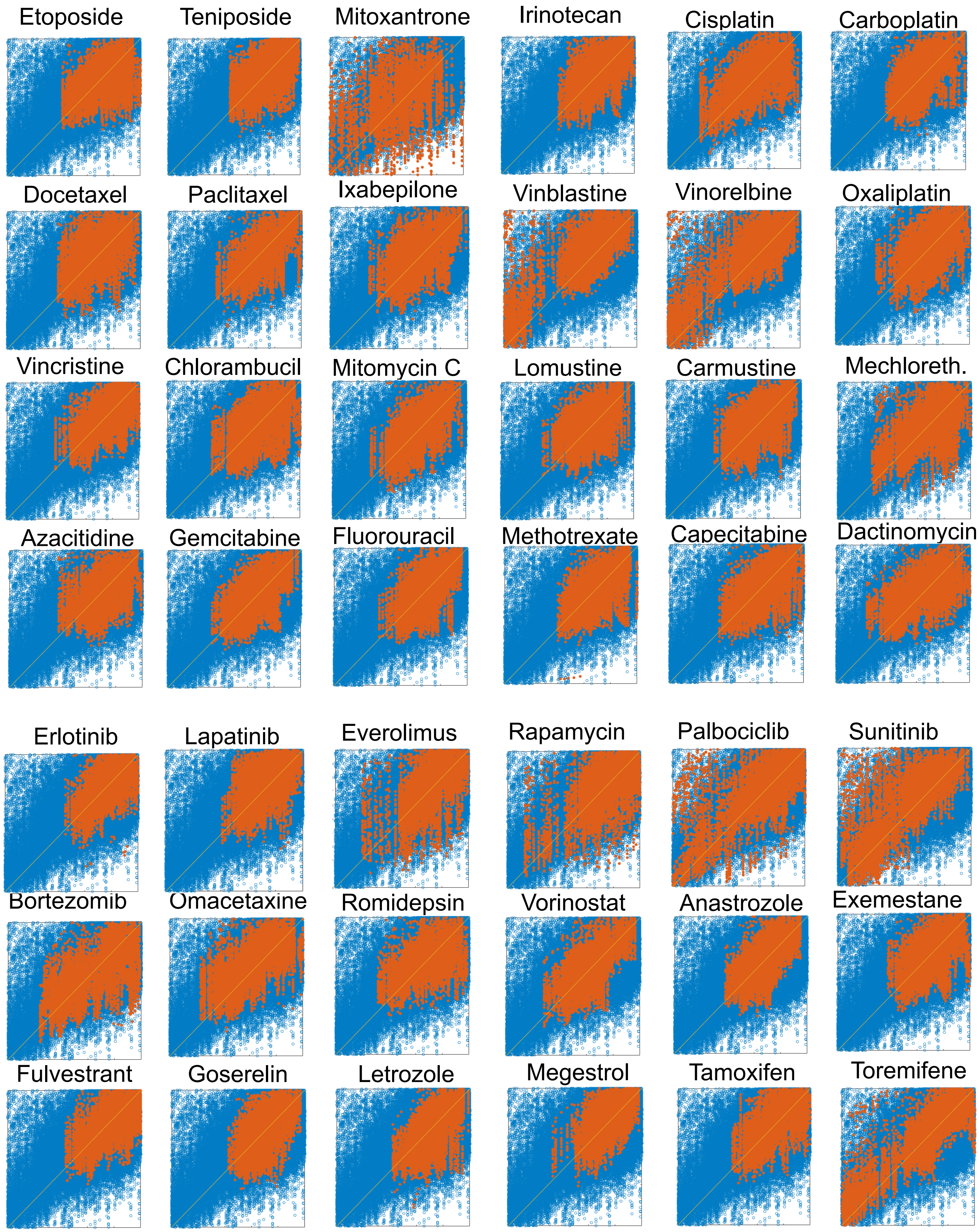

Appendix Figure S5: Nearly all classes of drugs are altered by TNBC-fibroblast interactions. Data plotted as in Figure 2E. Drugs organized by class, with cytotoxic agents on top (first 4 rows) and targeted therapies below (bottom 3 rows). Data for each drug are highlighted in orange.

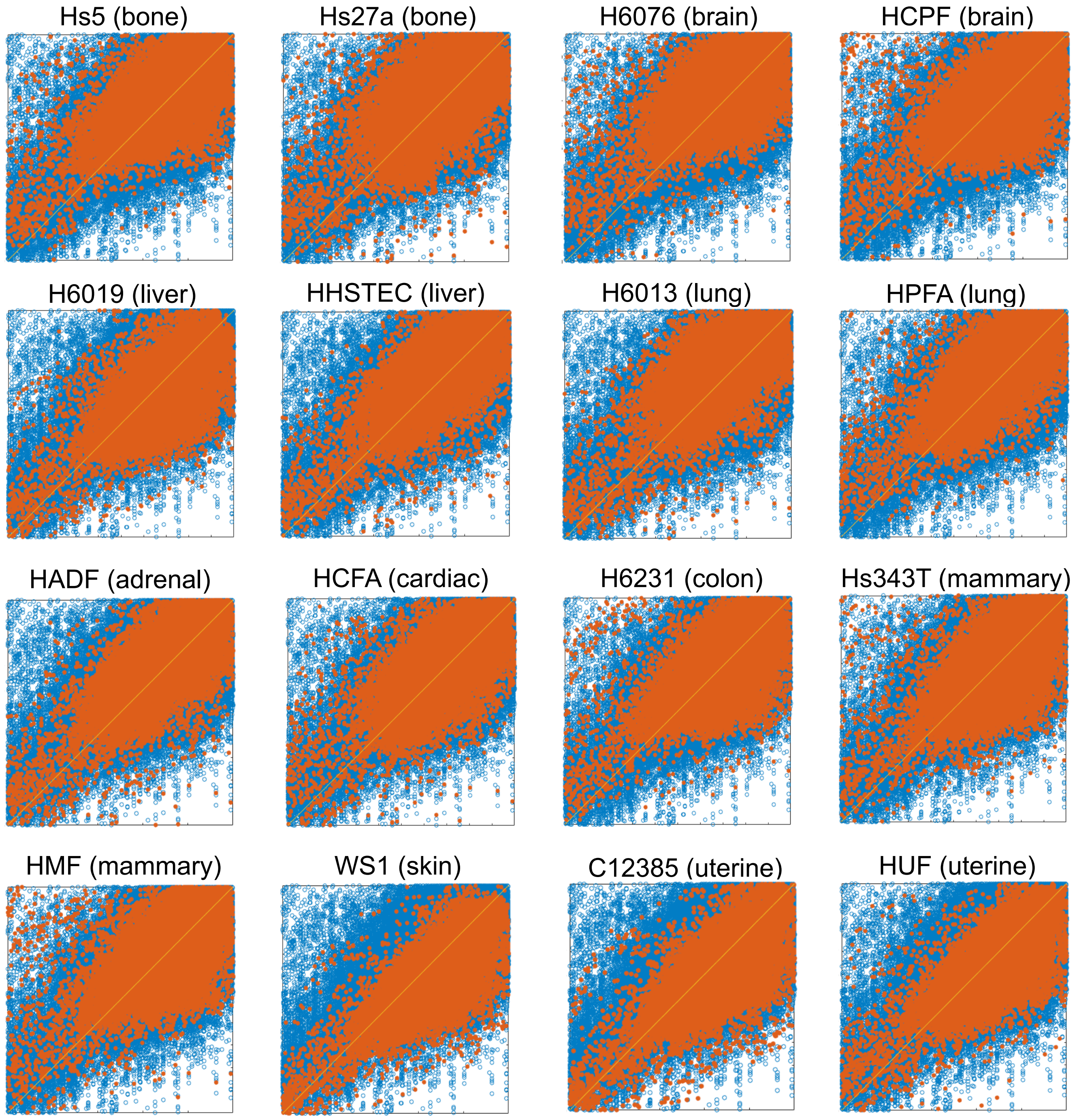

Appendix Figure S6: Fibroblasts specific modulation of drug responses in TNBC cells. Data plotted as in Figure 2E. In each plot, the overall dataset is shown in blue and the data for each primary fibroblast are shown in orange.

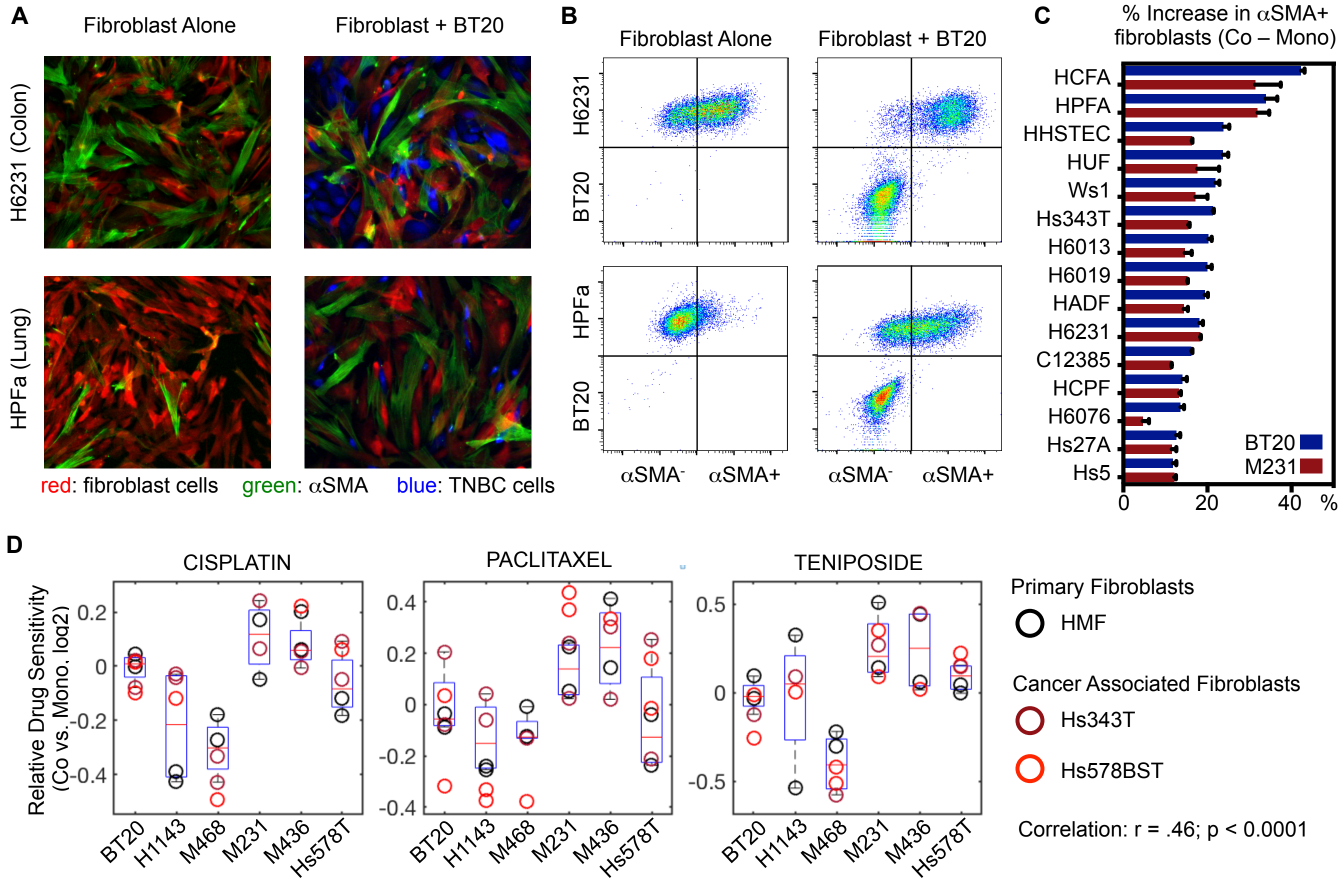

Appendix Figure S7: Primary fibroblasts grown in culture display an activated phenotype, similar to Cancer Associated Fibroblasts (CAFs). (A) Representative images of SMA expression (a marker of activated fibroblast state) in fibroblasts grown alone or in co-culture with BT-20 cells. SMA positive cells are green. BT-20 cells are labeled blue, and fibroblasts are labeled red, each using CellTracer dyes. Co-culture generally increases SMA expression. **(B-C)** Flow cytometry analysis of SMA expression. Representative FACS plots in (B). Data in (C) are the percent difference in SMA+ cells in co-culture relative to mono-culture for each fibroblast cell screened. The median % SMA+ fraction for fibroblasts in alone was 63% (range 10 – 85%). **(D)** Comparison of primary fibroblasts and CAFs. TNBC cells were co-cultured with primary breast fibroblasts or CAFs from breast cancers, and relative sensitivity to common anti-cancer drugs was quantified following 48-hour drug exposure. Boxplots represent the co-culture screening data as in Figure 2. Dots show relative changes in drug sensitivity induced by co-culture with indicated primary or CAF cells. Overall correlation between primary and CAF phenotypes was 0.46 ($p < 0.0001$).

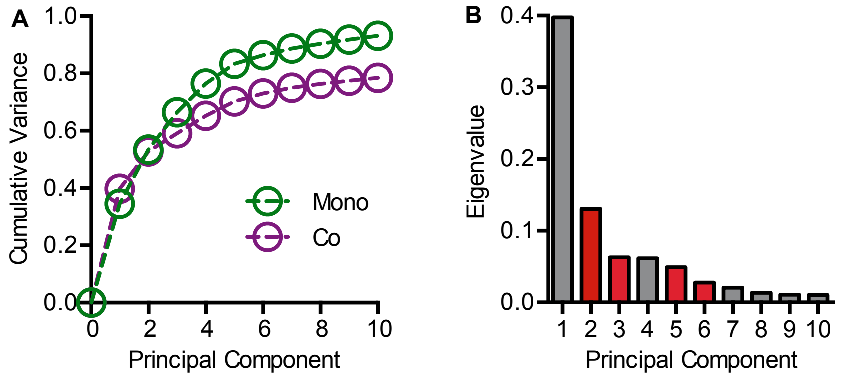

Appendix Figure S8: PCA of TNBC drug responses in co-culture (A) Cumulative variance (sum of eigenvectors) captured by PCA of mono-culture and co-culture drug responses. **(B)** Eigenvalues, which report the percent of total data variance captured by a given PC. Highlighted red, are PCs that are associated with the BL/ML dichotomy.

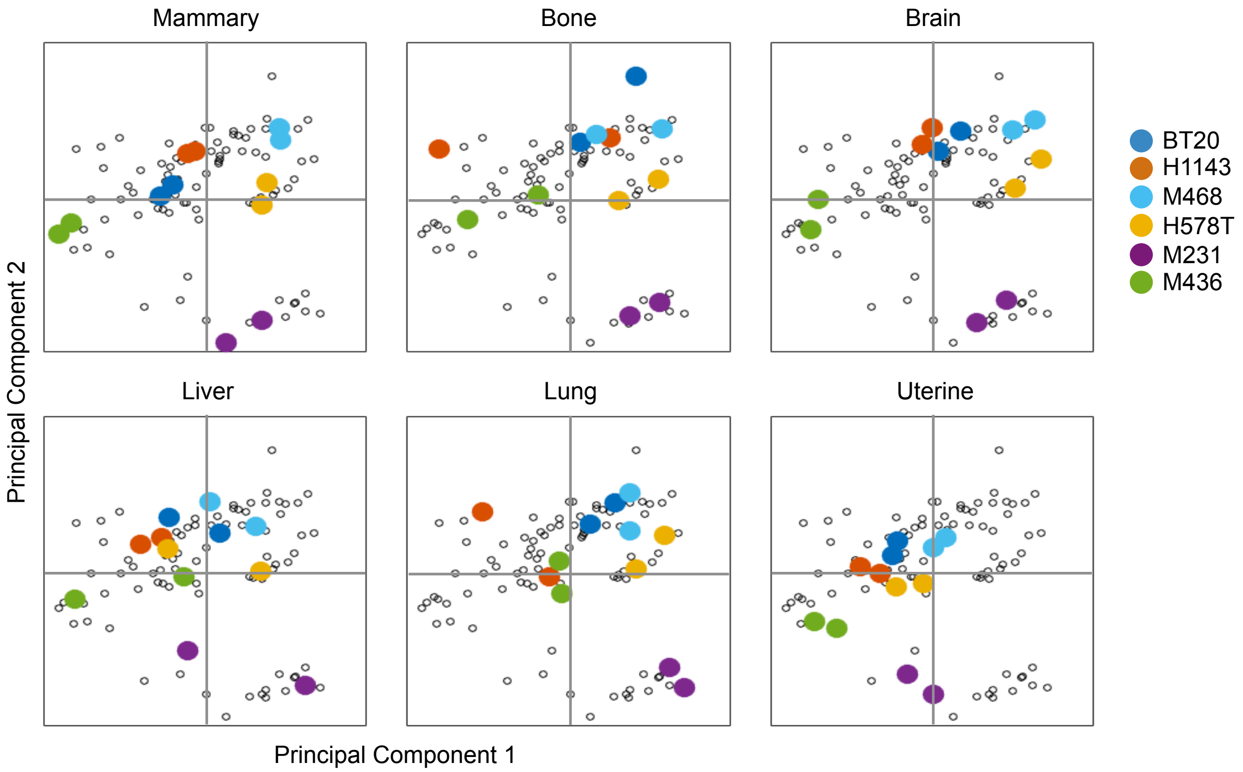

Appendix Figure S9: The first principle component captures variation associated with fibroblast tissue of origin. PCA scores projection, as in Figure 3A for six organs that were represented in our screen by multiple fibroblasts. In each plot, co-cultures are colored by cell line to highlight visually that fibroblasts from the same tissue/organ are more similar to each other than to random pairs. Statistical analysis shown in Figure 4B.

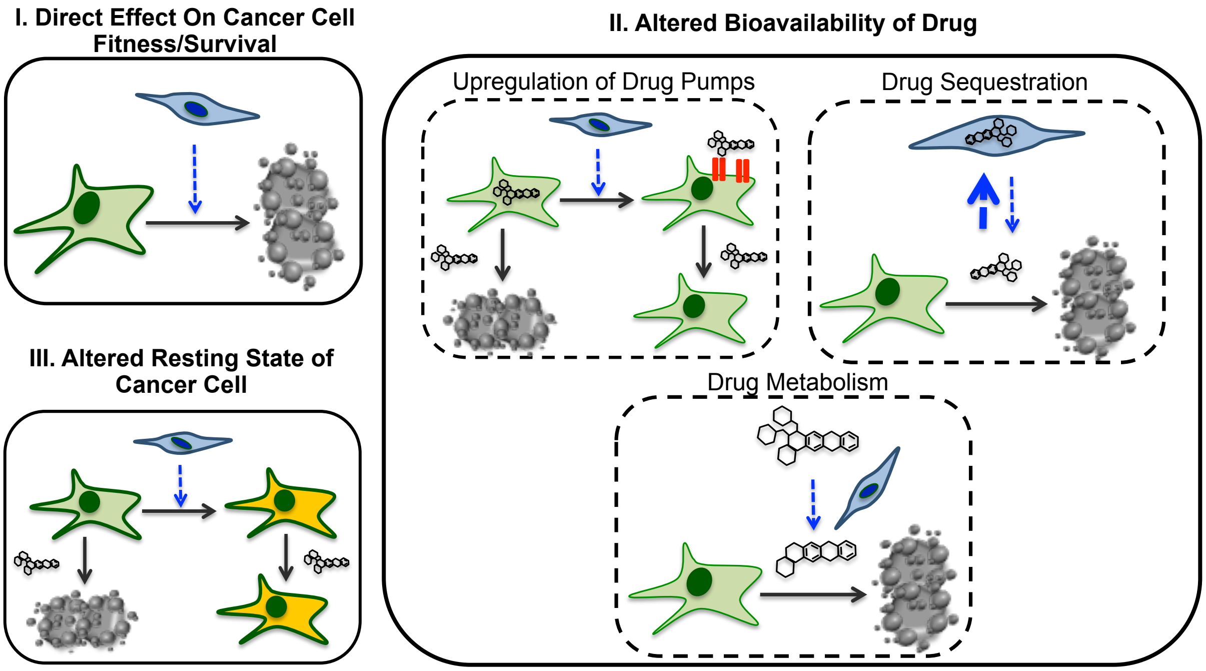

Appendix Figure S10: Conceptual mechanisms by which fibroblasts could alter broad-spectrum drug sensitivities of associated cancer cells. Overview of three conceptual mechanisms that were tested for how fibroblasts influence TNBC drug responses. A critical consideration is that our data show similar influence of a given fibroblast across many unrelated classes of drugs. Fibroblasts could I) directly kill some proportion of cancer cells, II) alter the availability or potency of drugs, or III) alter the resting state of cancer cells. In each diagram, fibroblast cells are blue, healthy cancer cells are green or yellow, and dead cells are grey.

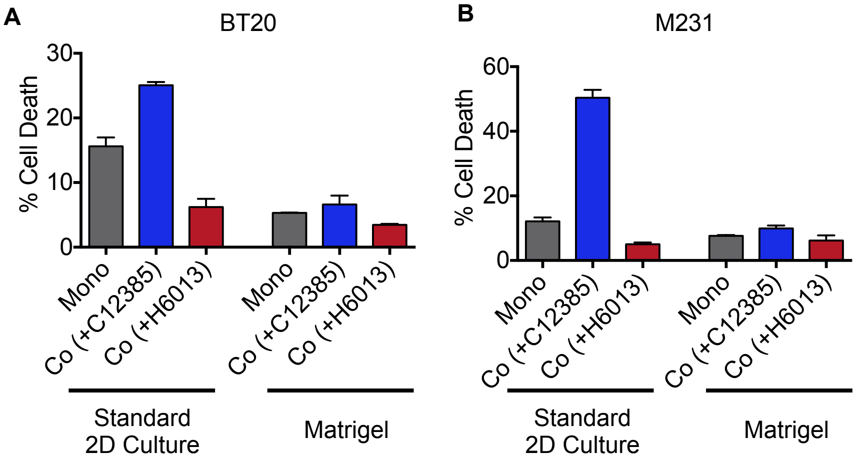

Appendix Figure S11: Growth in matrigel desensitizes TNBC sensitivity to doxorubicin regardless of the fibroblast co-culture environment. (A and B) TNBC cell death following exposure to doxorubicin measured by flow cytometry from cells grown in standard 2D culture or on matrigel coated plates. Matrigel reduces sensitivity to doxorubicin in both basal-like (BT-20, panel A) and mesenchymal-like cells (MDA-MB-231, panel B). Notably, drug sensitizing effect of C12385 was not observed in cells cultured with matrigel, suggesting that these phenotypes may not be seen when using *in vivo* xenograft protocols that require matrigel for tumor/fibroblast seeding.
